# A Qualitative Study of Stakeholders’ Views on Pharmacovigilance System, Policy, and Coordination in Pakistan

**DOI:** 10.3389/fphar.2022.891954

**Published:** 2022-06-09

**Authors:** Muhammad Akhtar Abbas Khan, Saima Hamid, Shahzad Ali Khan, Mariyam Sarfraz, Zaheer-Ud-Din Babar

**Affiliations:** ^1^ Health Services Academy Islamabad, Islamabad, Pakistan; ^2^ Fatima Jinnah Women University, Rawalpindi, Pakistan; ^3^ Center for Pharmaceutical Policy and Practice Research, Department of Pharmacy, School of Applied Sciences, University of Huddersfield, Huddersfield, United Kingdom

**Keywords:** pharmacovigilance, adverse drug reactions, views, perceptions, regulation, coordination, public health, Pakistan

## Abstract

**Objectives:** Due to the absence of necessary rules, poor coordination, and various challenges, the pharmacovigilance system of Pakistan is not optimally functional at all levels of the health system. The objective of the study was to assess the stakeholders’ perceptions of the current ADR reporting system and to identify the pharmacovigilance policy issues and problems of effective coordination.

**Methodology:** Stakeholders from a broad range of disciplines, academia, regulatory authorities, the pharmaceutical industry, international health organizations, as well as pharmacovigilance experts, and healthcare professionals were included in the study. A total of 25 stakeholders throughout Pakistan were interviewed during exploratory semi-structured interviews. The interviews were recorded digitally, transcribed, coded, compared, and grouped according to their similarity of themes. Participants provided insights into gaps, limitations, and challenges of Pakistan’s current ADR reporting system, issues with proposed pharmacovigilance rules, and coordination difficulties.

**Results:** The majority of the participants considered the ADR reporting system in Pakistan to be improving but in a nascent phase. The identified gaps, challenges, limitations of the system, and barriers to reporting were labeled as reasons for limited functioning. Almost all stakeholders were aware of the existence of draft pharmacovigilance rules; however, participants in the industry were familiar with the contents and context of draft pharmacovigilance rules. Bureaucratic red tape and lack of political will appeared to be the top reasons for delaying the approval of the pharmacovigilance rules. Wider consultation, advocacy, and awareness sessions of policymakers and HCPs were suggested for early approval of rules. Participants unanimously agreed that the approval of rules shall improve the quality of life and reduce the economic burden along with morbidity and mortality rates. The need for greater and collaborative coordination among the stakeholders in promoting medicines’ safety was highlighted. All participants suggested the use of media and celebrities to disseminate the safety information.

**Conclusion:** Participants showed partial satisfaction with the way pharmacovigilance in Pakistan is moving forward. However, stakeholders believed that engagement of multi-stakeholders, approval of pharmacovigilance rules, and the establishment of pharmacovigilance centers in provinces, hospitals, and public health programs (PHPs) shall support in achieving the desired results.

## Introduction

The thalidomide incident in 1961 marked a paradigm shift in the field of medicine safety (Fornasier et al., 2018). The World Health Assembly during its 16th session in 1963 adopted a resolution (WHA 16.36) that reaffirms the need for rapid dissemination of information regarding adverse effects resulting from medicines. This resolution paved the path to the formation of the World Health Organization Programme for International Drug Monitoring (PIDM) (Pal, 2013).

In 1978, the Uppsala Monitoring Center (UMC) was established to support the PIDM. All member states sent the individual case safety reports (ICSR) to the central database called VigiBase (UMC, 2022). The UMC is responsible to manage and maintain the VigiBase. It is a database with more than 28 million safety reports. The basic idea behind establishing this center was to collect adverse reaction reports from multiple sources across the globe to identify potential hazards related to medicine safety (UMC, 2018; WHO, 2022).

A national pharmacovigilance regulatory framework is considered an integral part of medicines policy in a country (Mehta et al., 2017). A set of rules, regulations, guidelines, and standard operating procedures are required for an efficient pharmacovigilance system to ensure medicine safety and data integrity. Similarly, the enactment of regulations ensures the legal cover for monitoring and compliance by all stakeholders (Nwokike and Eghan, 2010). The lack of a pharmacovigilance policy is seen as a contributory factor that medicines’ safety and quality may be compromised (Rasheed et al., 2019). The thalidomide disaster pointed out the inadequate regulations and the flaws in the regulatory processes adopted by the regulatory agencies. As a result, several countries have therefore introduced new legislation to reinforce their existing drug safety systems (WHO, 2002; Rice, 2007; Lembit and Santoso, 2010; Beninger and Ibara, 2016). The United States Food and Drug Administration in 1962 introduced the amendments which require safety and efficacy data on medicines prior to the premarketing submission. The United Kingdom introduced the Yellow card scheme to report suspected ADRs by healthcare professionals in 1964. Patients had access to submit yellow cards since 2005 (MHRA, 2022). In 1965, the European Union developed its first legislation applicable to its member states. A pharmacovigilance system at the EU level was established in 1995 and last strengthened with further regulations were implemented in 2012 which was a revolutionary step in the field of medicines regulation (Bahri and Arlett, 2014; EMA, 2022). These regulations strengthened transparency, stakeholders’ engagement, and safeguarding of public health. However, the regulatory framework and pharmacovigilance activities are not harmonized across various countries. Hans and Gupta (2018) found inconsistency and variance among regulatory functions of the United States, Canada, the United Kingdom, and India (Hans and Gupta, 2018).

The drug regulatory authorities around the globe have introduced user-friendly online ADR reporting systems including USFDA MedWatch (FDA, 2022), Yellow Card Scheme in the United Kingdom(MHRA, 2022), and mobile applications to effectively identify and address serious drug-related problems. The studies have shown that these systems are underutilized due to their voluntary nature in reporting ADRs (Hazell and Shakir, 2006). In the United States, less than 10% of ADRs are reported through MedWatch (Lasser et al., 2002). The countries which introduced patient reporting earlier, that is, the Netherlands, Denmark, and the United Kingdom, showed high reporting rates, while countries that introduced patient reporting recently, including Hungary, Portugal, and Malta, have low levels of ADR reporting (Inácio et al., 2017).

Similar to the thalidomide disaster, the Isotab tragedy in Pakistan highlighted the importance and need for introducing an ADR reporting system at all levels of healthcare establishments (LHC, 2012). The use of contaminated cardiac medicine took the lives of more than 300 patients. The judicial inquiry tribunal (JIT) established to determine the causes of deaths in the Punjab Institute of Cardiology, Lahore, observed that there was no system of ADR reporting in the hospital and supplier firm. It was further found there is no pharmacovigilance system that exists in the country. The JIT recommended introducing a system of yellow slips for reporting ADRs to the hospital committees set up for the said purpose. Moreover, it was also suggested to set up pharmacovigilance centers at the level of the health department to process and share information regarding drug reactions and other related matters with health professionals and hospitals (LHC, 2012). In 2015, Drug Regulatory Authority of Pakistan (DRAP) established the national pharmacovigilance center. This was in order to collect the ADR reports from all stakeholders (Qato, 2018). Consequent to this, DRAP became a full member of the UMC in 2018 (WHO, 2022). A study (Khan et al., 2022) revealed that the pharmacovigilance system in Pakistan is not fully functional at all levels. Presently, ADR reporting is voluntary. Currently, there is a med vigilance E-reporting system (DRAP, 2018) and Web-RADR med-safety mobile application for reporting ADRs (DRAP, 2020). However, the collected number of ADRs related to medicines is not sufficiently corresponding to the population of the country (Syed et al., 2018; Khan et al., 2022). A recent study also identified gaps in the pharmacovigilance system including the absence of the pharmacovigilance legal framework that will require mandatory ADR reporting by the stakeholders (Khan et al., 2022).

In Pakistan, majority of the physicians are not aware of the ADR reporting system, and there is inadequate coordination between the physicians and other healthcare professionals (Hussain et al., 2020b) and other stakeholders (Khan et al., 2022). Some studies have investigated only the barriers to ADR reporting (Hussain et al., 2018, 2020a, 2020b; Nisa et al., 2018; Syed et al., 2018), while no study has been conducted to explore the other issues related to the ADR reporting system, pharmacovigilance policy and legal framework, and stakeholder’s coordination.

This study aimed to fill this gap and explore the multi-stakeholder views and perceptions about the pharmacovigilance system in Pakistan. The study also aimed to explore the pharmacovigilance stakeholder’s opinions and perceptions regarding challenges, barriers, limitations, and the gaps related to the ADR reporting system in the country.

## Methodology

### Study Design

Through an inductive qualitative approach (Thomas, 2006), in October–December 2021, the study was conducted using semi-structured interviews (Kaae and Traulsen, 2015). A deductive approach was applied to frame the interview guide questions.

### Participant Selection

A purposive sampling technique was used for this study (Campbell et al., 2020). A list of potential participants for the study was prepared from various fields including present and former federal ministers, bureaucrats (senior officers in Ministry/Health Department), and technical officers working in the federal and provincial drug authorities, academia, experts on medicine safety and pharmaceutical policy and practice, pharmaceutical industry (multinational and national), physicians, and nurses (Table 1).

**TABLE 1:** List of stakeholders contacted and participated in the study.

Stakeholders	Stakeholders contacted and invited (n)	Stakeholders who accepted the invitation (n)
Federal ministers (current and former)	3	0
Bureaucrats/civil service officers (federal and provincial)	2	0
Government officers as pharmaceutical regulators (federal and provincial)	11	6
Academia, pharmacy/medical (public and private sector)	4	3
Pharmacovigilance consultants	5	4
Pharmaceutical practice and policy expert	1	1
Representatives of international health organizations	2	2
Physicians	2	2
Nurses	2	1
Public health program	1	1
Pharmaceutical industry (multinational and national)	5	5
Total	38	25

The inclusion criteria included: a) participants working or involved in Pakistan’s healthcare system (doctors, pharmacists, and nurses); b) participants having a current or minimum of 5 years of experience or involvement in the policy development and ADR reporting or medicine safety activities; c) participants who were fluent in the English language. The participants represent the larger sample of all the persons involved in pharmacovigilance in Pakistan. The participants were recruited through phone calls, WhatsApp messages, and emails. Thirty-eight participants were contacted, out of which 35 responded to the invitation. Three participants did not reply to the email and subsequent reminders. Five participants initially agreed to participate but later showed reluctance to record the interviews. Furthermore, five participants had issues with the availability of time for the interview. This resulted in 25 participants.

Information sheets and consent forms (see supplementary material) were sent to the participants who gave consent for the interview. The range of duration of the interviews was between 16 and 55 min. The mean interview time was 33 min.

### Interview Guide Development

We conducted a comprehensive literature review to determine the existing knowledge about the current ADR reporting system, pharmacovigilance policy/rules/regulations, and coordination among stakeholders. The literature search was conducted by using the keywords “pharmacovigilance, adverse drug reactions, ADR, policy, regulation, qualitative study, policy analysis, coordination, and stakeholders” on search engines such as Google Scholar, ScienceDirect, HINARI, and PubMed. This literature review fed to develop the guide (Ritchie and Lewis, 2003; DiCicco-Bloom and Crabtree, 2006; Guion and Mcdonald, 2006; Turner, 2010; Babar et al., 2012; Babar and Francis, 2014; Hussain et al., 2018, Hussain et al., 2020b; Phillips et al., 2021; Khan et al., 2022).

The following broad themes were identified, and subsequent sets of questions were developed. These included 1) perception of the current ADR reporting system in Pakistan, including participants’ awareness, understanding, opinions, and views on challenges, gaps, limitations, barriers, and approaches for improvement; 2) role of the pharmaceutical industry in the promotion of medicine safety; 3) future research needs; 4) views on draft pharmacovigilance rule, participants’ awareness, understanding, opinions, and impact on public health; 5) perception on coordination, stakeholder engagement and communication; gaps in stakeholders selection, placement of the National Pharmacovigilance Center, and DRAP are aiming to promote public health and the issues related to unethical medicine promotion by the pharmaceutical industry; and 6) the role of media in promoting medicine safety (Table 2).

**TABLE 2:** Themes and sub-themes.

Theme	Subtheme	Details
General views on the ADR reporting system of Pakistan	Understanding of the current ADR reporting system	Perceptions about gaps, limitations, challenges, and barriers to the ADR reporting system
Views to improve the current pharmacovigilance system
Future pharmacovigilance research needs in Pakistan	Future ideas on pharmacovigilance research requirements
Role of the Pharmaceutical industry in the promotion of medicines' safety	Knowledge about pharmacovigilance activities conducted by the pharmaceutical industry	—
Views on draft pharmacovigilance rules in Pakistan	Familiarity and understanding of the issues of draft pharmacovigilance rules	Knowledge of draft pharmacovigilance rules
		Factors involved in delaying the approval of the pharmacovigilance rules
		Expediting the approval process of pharmacovigilance rules
		Impact on public health and medicine safety after implementation of pharmacovigilance rules
Coordination, stakeholder engagement, and communication	Need for greater harmonization	Description of coordination between DRAP and other stakeholders
		Explanation of personal experience of contacting DRAP for safety information
		Knowledge sharing and stakeholder engagement
Gaps in the selection of effective stakeholders	Identification of key stakeholders to improve the pharmacovigilance system
	Placement of national industry
	Pharmacovigilance center
Media and medicine safety	Role of media in medicine safety promotion	Selection of media for medicine safety promotion

The interview guide was tested for its validity and reliability by two experienced researchers at the Health Services Academy, Islamabad, and Quaid-i-Azam University Islamabad, Pakistan. The interview guide was piloted by one pharmacist from the Drug Regulatory Authority of Pakistan (DRAP) and another one from the World Health Organization with involvement in policy development and the ADR reporting system.

After the verbal consent, an information sheet with a consent form (see supplementary material) was sent to the participants through email. The interviews were conducted on Zoom video conferencing (https://zoom.us/) and were recorded after permission by the respondents. The interviews were transcribed verbatim (space fillers were omitted). Both participants were sent their audios and transcripts to edit and approve. The interview guide was amended after the pilot interviews (see appendix-A supplementary material). One question was deleted, and three questions were added based on information received from the respondents.

### Data Collection

**FIGURE 1:**
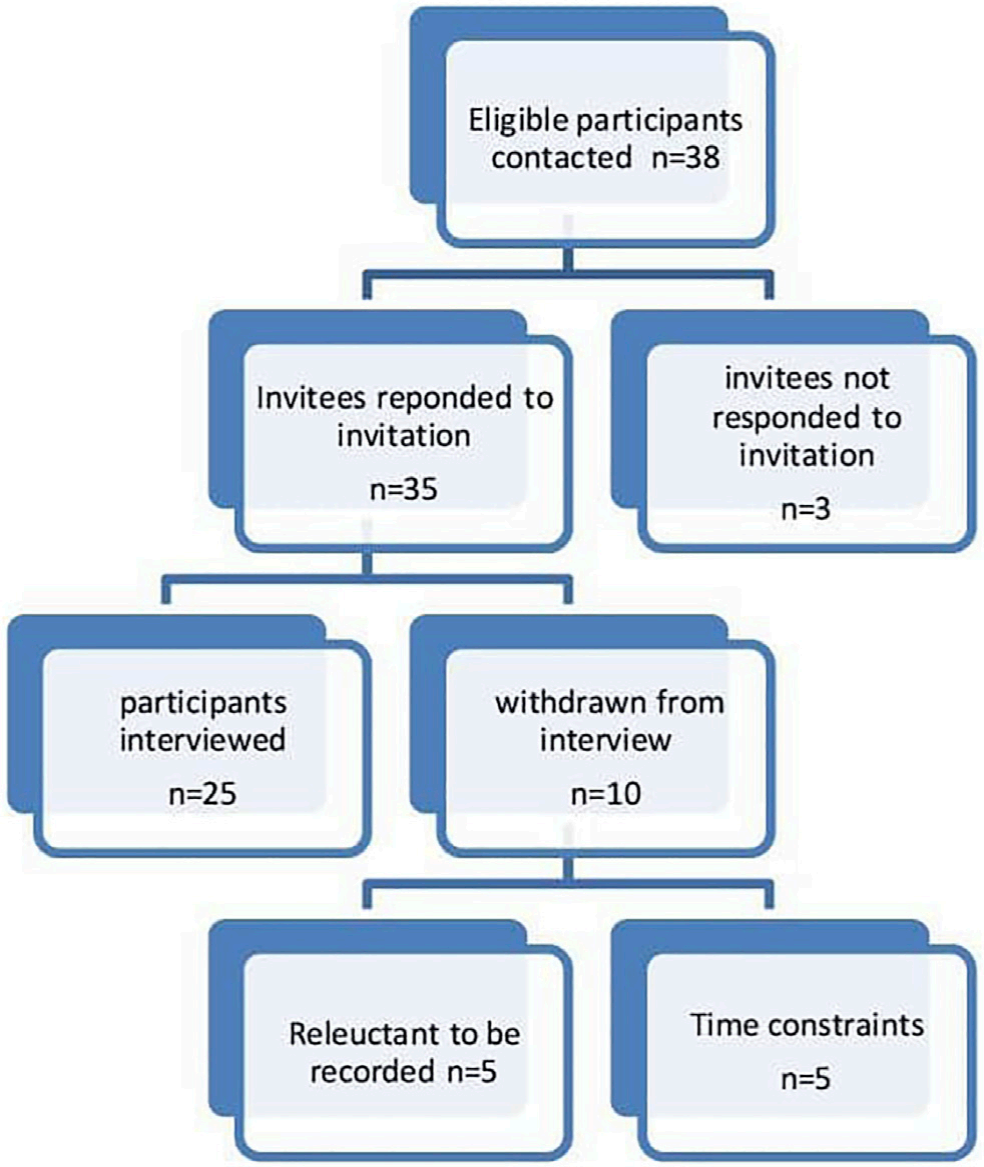
Flow diagram of participants’ selection.

Twenty-five stakeholders were interviewed (Figure 1). Ten interviews were conducted in person, 14 on zoom video conferencing, and one on a mobile phone. Before conducting the interviews, the participants were briefed on the study and were informed that interviews are voluntary and they have the right to withdraw from the interview at any time. Consent was taken before the recording of the interviews. All interviews were conducted in the English language. The interviews were recorded on mobile phone and Zoom video conferencing application and saved on a password-protected computer. Coding was carried out on the interviewees to ensure anonymity (Table 3). No financial compensation was offered to the participants. The interviews were transcribed intelligent verbatim (McMullin, 2021).

**TABLE 3:** Stakeholder’s characteristics: Stakeholders *n* = 25.

Stakeholder	Designated in thesis
Federal government service	FGS-I
	FGS-II
	FGS -III
	FGS–IV
Provincial government service	PGS-I
	PGS-II
Academic pharmacy	AP-I
	AP-II
Academic physician	APhy-I
Physician	Phy-I
	Phy-II
Pharmacovigilance experts (PE)	PE-I
	PE-II
	PE-III
	PE-IV
Pharmaceutical policy and practice expert	PPPE-I
Pharmaceutical industry (PI)	PI-I
	PI-II
	PI-III
	PI-IV
	PI-V
International Health Organization (IHO)	IHO-I
	IHO-II
Nurse	N-I
Public health program	PHP-I

### Data Analysis

Thematic analysis was performed according to the process explained by Braun and Clark (Braun and Clarke, 2012). A team of experts performed a staged analysis of the interviews. Initially, transcripts were read thoroughly to take notes and to record the key themes and codes. Subsequently, a basic coding framework was developed outlining the subthemes, categories, codes, and quotes. In the last stage, the group of researchers further refined the codes.

## Results

A total number of 30 participants showed willingness to participate in the research, and 25 interviews were conducted. Demographic information including age, gender, profession, and length of experience was also recorded (Table 4). The majority of the participants (*n* = 16) were pharmacists by training: one-fourth were medical doctors (*n* = 8) and one was a nurse. More than 80% participants (*n* = 21) were male, while less than 20% (*n* = 4) were female. Out of 25, eight participants were government employees, 12 were from private organizations, and five were consultants. The results are listed as follows.

**TABLE 4:** Stakeholders’ characteristics.

Details	Number of participants
Public sector employees	8
Private sector employees	12
Freelance consultants	5
Profession	
Doctors	8
Pharmacist	16
Nurse	1
Gender	
Male	21
Female	4
Age of participants	
<40	7
40–60	13
60 +	5
Experience (years)	
10>	5
10–20	5
20 +	15

### Theme No. 1: General Views on the ADR Reporting System of Pakistan

The majority of the participants felt that the ADR reporting system in Pakistan is evolving but it is in its infancy. Some participants thought that the pharmacovigilance system is only limited to tertiary care hospitals and immunization programs.

“To be honest with you, the ADR reporting system in Pakistan has been in a transition … the ADR system which is in process at the moment looks very potential very promising. And we will only know about its impact once it starts to take hold and starts to grow roots in the system, which may not be the case at the moment (PPPE -I)”.

Presently, 50 to 55 firms are reporting ADRs, and 60% of reports are coming from the expanded program for immunization. The perception of a few participants about DRAP had changed during the COVID-19 vaccine roll-out. A participant from the public health program acknowledged the good work of DRAP during the pandemic.

“Our views have basically strengthened or we have our perception has been cleared during the COVID vaccination rollout. Before that, we were not clear about how was DRAP working or how were we supposed to report to DRAP (PHP-I)”.

The absence of a proper pharmacovigilance regulatory framework is identified as the major gap in the ADR reporting system. Almost all participants mentioned that without the implementation of pharmacovigilance rules how one can expect stakeholders to report ADRs. A policy expert was “not satisfied the way it has been handled” (PE-III).

According to a pharmaceutical policy and practice expert, “the biggest gap would be the reporting by the health system into a national database” (PPPE-I). The connection between hospitals, community pharmacies, and central and provincial pharmacovigilance centers is missing. A physician stated that he was aware of some vaccine monitoring networks that keep track of adverse events (AEs) associated with their vaccines as a requirement of principle manufacturers, but they are not integrated with the national system. A similar statement of a tertiary care hospital nurse is as follows:

“We actually report different medical errors, different adverse reactions, and all these things to our control system for HMIS. And then this will go to the quality department and then I don’t know where'd they go? That I don’t know (N-I)”.

Few participants thought that the absence of a causality assessment committee and not having the capacity of the national pharmacovigilance center to evaluate individual case safety reports (ICSRs) are essential gaps in the system.

“They have extensive AEFI reporting systems; at least for this COVID vaccination, they have 50,000 AEFI data, but the capacity of the DRAP to collect these data and generate the safety signals that is also lacking. (IHO-II)”.

Several participants commented that pharmacovigilance should be included in the curriculum of medical and pharmacy undergraduate degree programs. A participant thought that the pharmacy’s curriculum does not address these issues (PE-III). He further added that community pharmacies have not been engaged in the collection of ADR reports.

“85% of our drug consumption is at the community pharmacies. And I'm not sure if they have been brought into the loop on this important element. I think mostly, we have been focusing on some hospitals in the country. (PE-III)”.

Underreporting is identified as a major limitation to the ADR reporting system in Pakistan by the participants. The factors which contribute to the under-reporting are lack of awareness and training of healthcare professionals (HCPs), education of patients and consumers, noncoordination between regional and national pharmacovigilance centers, lack of communication among HCPs, no or limited private sector engagement, lack of information on drug exposure, mistrust on the system, no use of technology or the resources through which the reports had to channelize to the centers, shortage of skilled human resource, no mandatory requirement of ADR reporting, and lack of training and the understanding of the ADRs. Many participants believed that filing an ADR report and receiving no response or feedback from the regulator is very discouraging for future reporting.

“It is not clear what is to be reported and where and then that feedback is never given back to the people, what happened to it and all so, those the two-way communication isn't there (Phy-II)”.

Two participants considered the devolution of the health sector in 2011 from the federal domain to the provincial domain, a challenge for coordination among federation and provinces regarding medicine safety. Another participant felt that regarding medicines safety there is “no active listening”.

“There is a system in the making of the ADR reporting system. I hope that would actually soon be smart enough and listening enough so that patient responses could be actually picked up early enough and completely enough. But at the moment, there’s no communication system, whereby reports could actually be communicated back into the system where it could be actually aggregated at a larger level (PPPE-I)”.

The participants talked about other challenges of the current ADR reporting system, including the weak surveillance system, reluctance in reporting, low quality of the reported data, and biases in reporting. Lack of awareness, communication channels, trained staff, infrastructure, and facilities, are additional challenges of the ADR reporting system. According to a federal regulator, fewer experienced technical staff is the reason for a lower number of reports sent to the global system. A participant informed that the pharmaceutical industry is reluctant to implement a pharmacovigilance system because they have no profit coming from this activity. An expert considered less understanding of patients and attendants as a primary challenge to the ADR reporting system because patients and their attendants are not able to distinguish between the symptoms of disease and adverse effects of the medicines.

“I think the primary challenge in ADR reporting comes from the one: the patient himself; ourselves, which where they may not actually be able to determine whether whatever is happening to them, if it is a strange phenomenon if that is because of intake of medicine, and then while reporting back, they may not be very clear about what their experience has been (PPPE-I)”.

For all participants who were working in hospitals or the pharmaceutical industry, the leading barrier to ADR reporting is the fear of punitive action, punishment, and regulatory action, fear of losing a job, and public protest in case any ADR happened to the patient. A former hospital pharmacist thought that a “lack of trust in the system” hinders the HCPs to report while a physician commented that there is a disincentive in reporting with a feeling that “if I shall report, I shall get caught” (Phy-I).

“The main barrier is that the person who is supposed to report is the person who’s administering the drug or the vaccine. So what happens is that they’re very scared of punitive action or anything that might go against them and reporting an ADR (PHP-I)”.

An industry representative mentioned that “doctors are absolutely very, very busy with their practice for a large number of patients. So they do not have time to report adverse events”(PI-III). A federal regulator considered the illiteracy of people and the language of reporting forms as the biggest barriers to reporting.

“The biggest barrier in Pakistan is, of course, due to low literacy rate is communication in language query using; the language used by the regulators or the HCPs is most often the official language English however, the patients are unable to understand these things in that language (FGS-I)”.

The participants suggested various interventions and strategies to improve the current ADR reporting system. Early approval of pharmacovigilance rules, the establishment of provincial pharmacovigilance centers and ADR reporting centers in hospitals, giving priority to the subject, training of HCPs, capacity building of hospitals and the pharmaceutical industry, and awareness of HCPs and patients are the most important aspects to improve the pharmacovigilance system in Pakistan.

Two participants suggested initiating the behavioral change communication strategies among all stakeholders to encourage the culture of reporting ADRs. An academic pharmacist recommended involving the religious clerics to advocate the ADRs reporting during Friday prayer sermons.

“Behavioral change communication, a lot of good campaigns, which would actually aim at consumers, doctors, paramedics, system operators, would need to be actually carried out so that they are willing to share information and other barriers (PPPE-I)”.

All stakeholders should adopt a joint strategy regarding the need to strengthen liaison and confidence among all. The infrastructure of pharmacovigilance centers in the industry requires improvement. Doctors and pharmacists should provide counseling and education to patients and relatives. There is a need to convince people on the grounds who are directly dealing with patients to report. One participant proposed media campaigns on medicine safety. Both physicians thought that there should be no punitive action against a person who has honestly reported the ADRs.

An academic pharmacist emphasized that for the promotion of the pharmacovigilance culture in the country, institutional purchase of medicines should be linked with the existence of a pharmacovigilance system. Few participants proposed the information and behavioral change exercises, the active role of civil society, and the training of provincial officers. An academic physician suggested developing the culture of community pharmacies in the country.

“I have seen in other countries that they have this facility extended right into their communities, the community pharmacies, their pharmacist, visits the homes of the patient, chronically ill patients, and provides them the proper advice about the safe use of the drugs. So that is something which we just can’t dream of, in this country (APhy-I)”.

A participant among the federal government officers recommended training the regulator and the HCP on the Urdu version of all terminologies related to ADR to remove the language barrier. He further suggested people should be sensitized about ADR reporting in their languages. Added to this, at least those companies which are introducing new medicines should appoint pharmacovigilance officers to make liaison with the HCPs and analyze the reported data.

One participant argued that DRAP should analyze the reports it receives and a federal government officer identified a missing link of the regulatory authority that is not publishing the received information.

“My advice to DRAP would be that please analyze those reports, after the company submits those reports, please analyze those reports (PE-III)”.

A federal regulator stressed the provision of a dedicated budget at every level of the healthcare system. He stated that funds are required for spreading awareness, training of HCPs, equipment, and human resources. There should be no frequent transfers of the employees working in pharmacovigilance departments.

A physician from the industry suggested that in hospitals, a team of doctors and pharmacists should work together in the pharmacovigilance department.

“We need to have a very you know, kind of way cross-pollinated system where pharmacists and doctors should have a very important team. The person who is working on pharmacovigilance should either be supervised by a doctor in any pharmaceutical company (PI-II)”.

### Theme 2: Role of the Pharmaceutical Industry in the Promotion of Medicine Safety

All participants believed that the pharmaceutical industry plays a key role in the promotion of the safety of medicines because it is their “social responsibility” to report about the safety of medicines. Many believed pharmacovigilance activities performed by the industry are limited to routine surveillance. Two participants commented that in Pakistan, multinational firms are reporting ADRs to DRAP because of their obligation toward their parent company while local companies are still not at par. Few participants argued that there is a disincentive for the industry in reporting because of the “huge investment,” “low understanding of the ADR reporting system,” and “no obligation” by the law. A federal regulator thought that the industry’s role is not more than 15% because HCPs and the public report to the regulatory authority not to the industry. Another federal regulator commented that funds are required to run the ADR reporting system while financial support required to collect such data is neither supported by the governments nor by the pharmaceutical industries. The limited information is not sufficient to take any regulatory decisions.

“Currently, almost 40 or 50 pharmaceutical companies are reporting to the DRAP, but the data is not so much what you can say so much big that you can take the decisions based on that data (FGS-II)”.

Few participants thought that the industry is only interested in profit-making. A physician while sharing his experience of attending the medical conferences said that the industry never shares a bad side of the medicine. His statement is as follows:

“If you want me a blank answer, they have a role, but they don’t follow a good roleplay. I have been dealing with so many companies, and they always come and praise about their medicine, they never tell you this, you know, this side effect (Phy-I)”.

A nurse thought that the pharmaceutical industry only “hires doctors” and never arranges educational seminars for the nurses, despite being important stakeholders. A doctor from public health program thought the pharmaceutical industry gives very little importance to telling the message.

“If you’ve had the chance to look at the ads, for an over-the-counter drug, there is only at the end of the ad, they have a very small slot for saying that all medicines should be kept away from children. They may have effects, side effects, or anything. But the thing is that that message is completely lost in the entire promotion of the medicine itself (PHP-I)”.

### Theme 3: Future Pharmacovigilance Research Needs of Pakistan

There is a limited collaboration between academia, industry, regulator, and HCPs regarding medicines’ safety. On enquiring from participants about future research needs of pharmacovigilance in Pakistan, one participant stated that the biggest need in Pakistan is how various study designs are developed and implemented.

“In many of the hospitals, you might have retrospective data on some reporting, but it has not been collected not has been studied in a cohort manner. Neither there is a regulatory obligation for that nor the industry is interested in that and the HCPs themselves do not perform such studies because of the lack of interest from their side because their interest is more on the clinical side. So, this is one thing that you need to establish ADR linked with the study design especially the active surveillance and the passive surveillance study design. This shall also be propagated through the academicians as well as HCP levels (FGS-I)”.

A public health expert talked about research on the off-label use of medicines. Two participants emphasized the need for local clinical trials and safety data. A physician said, “if research is done in other parts of the world, it does not mean that the same research is effective on our population” (Phy I). A pharmaceutical industry representative pointed out that the local medicine safety newsletters contain only information related to international signals and product quality issues. The information is not from Pakistan in the local aspect, and all of the signals or the box warnings are from the international data. The statement of the academic physician is as follows.

“Yes, particularly, the local data is very, very important, because with the new medicines, which are being introduced, now, the importance of genetic factor is becoming more and more important. So, we just cannot rely on the data of other countries, we have to have our own data as well. So, if we have this data available, this will help us to make our own guidelines. And we can also issue instructions about the safe use of these drugs (APhy-I)”.

### Theme 4: General Views on Draft Pharmacovigilance Rules in Pakistan

The majority of the participants (*n* = 22) were aware of the existence of draft pharmacovigilance rules except for three participants: two physicians and one nurse did not know about the existence of such rules.

Most of the participants accepted that they have seen the initial drafts and have not reviewed them recently. The reasons they explained were that the “rules were shared long ago” and “no new stakeholder consultation” was arranged by the regulator. Only the participants from the pharmaceutical industry were aware of the context and contents of the rules. A pharmacovigilance expert said:

“I’ve come across really, but I have not gone through the very fine tooth comb scape? I have not looked at line by line, but I think I’m reasonably aware of it. Yeah. If you ask me, have you read it? My answer would be no (PE-III)”.

There were conflicting opinions among the participants if the draft rules are aligned with the international best practices. The current and former employees of multinational pharmaceutical companies found the rules aligned with the international standards with no shortcomings. According to them, draft rules are adapted from EMA and FDA regulations and WHO guidelines. The participants from federal government services also believed the rules are drafted as per international practices. Some participants argued that rules are adapted from international regulations and are not made in the local context. A statement of an expert is as follows:

“They might be aligned with international standards. So, but that’s the cut and paste situation. But are they relevant to our country? I have my doubts about that (PE-III)”.

Some participants pointed out that the rules are complicated, and they do not define the roles and responsibilities of stakeholders. One participant from the pharmaceutical industry suggested that a qualified person for pharmacovigilance should be free from “commercial bias.” A participant from the federal government service informed that to ensure transparency, an independent chairman of the risk assessment committee (not from DRAP) has been proposed in amendments to the rules. Few stakeholders thought that shortcomings cannot be pointed out, and rules cannot be improved without implementing them.

“Rules cannot be improved until they are implemented. Once implemented limitations come and with the passage of time to know the problems hurdles in these rules, and that’s why with the passage of time amendments are made in the rules to make them better and better (FGS-II)”.

A pharmacovigilance expert who is also a pharmacist sees bias and conflict of interest in the whole system. He thought that rules are drafted by the pharmacists, and the objective of the whole exercise looks to promote pharmacists, not patients’ safety. The participants from international health organizations suggested wider dissemination of the rules before approval and include the role of the healthcare commissions in the proposed rules.

Almost all stakeholders rated “bureaucratic red tape” as the top reason for delaying the approval of the pharmacovigilance rules. One participant believed that the bureaucracy does not understand the importance of the issue.

“I think that it might be a bureaucratic red taping because if it were, it had been drafted in 2017 and now it is 2021 and still it hasn’t been notified (FGS-III)”.

Many participants stated the “lack of political will” for the delay in the approval of pharmacovigilance rules. They think that the government is not clear in taking steps and that its commitment to medical safety is not there. Several participants mentioned that there is no willingness from stakeholders, that pharmacovigilance is not on the agenda, and policymakers are not competent. The pharmacovigilance job is usually assigned as additional work to the officers in provinces and hospitals or given to the junior and inexperienced officers and it usually does not work. One participant thought that pharmacovigilance is not the priority of the policy and decision-makers.

“The only and only thing is that, as I already shared with you, that pharmacovigilance system and ADR reporting is never, never a priority for any of us, for our policymakers, for the people who are involved and who are at the helm of affairs in the health ministry, even DRAP everywhere (PGS-I)”.

Two participants doubted the immediate implementation of the pharmacovigilance rules due to lethargy and the capacity of the system. Some stakeholders think that it shall be an economic burden on the industry to set up the pharmacovigilance system and hire the services of qualified pharmacovigilance experts.

“…Pharmaceutical companies also don’t like these rules to be implemented, because it’s a burden for them as well in an implementation that really related to the resources related to the system related to the implementation overall(PI-III)”.

On questioning how the process of approval of pharmacovigilance rules be expedited, some participants proposed to arrange formal consultations of all stakeholders, giving it a priority and setting the timelines. Several participants believed to initiate advocacy, as well as sensitizing the political leadership and bureaucracy.

A participant suggested that leadership be sensitized to pharmacovigilance to achieve WLA (WHO listed authorities) status. The participants from the pharmaceutical industry believed that the involvement of trade bodies can strengthen the proposed rules.

“Hopefully, we are going for the WLA (WHO listed authorities) and in these aspects rules, approval of rules can be accelerated because the higher management should show the commitments that DRAP shall achieve the WLA and WLA is not possible without promulgation of pharmacovigilance rules. It is one of the basic requirements and it is the level one indicator (FGS-II)”.

Most participants were confident that approval and implementation of pharmacovigilance rules shall not only ensure early detection of medicine-related risks but also can minimize their harm, can reduce morbidity and mortality rates, as well as the economic burden.

“If pharmacovigilance system starts to take hold in Pakistan and if the reports coming back, properly analyzed, if the issues are being identified, and that would be the start. And if after that, you can work backward, to prevent ADRs on a larger scale. So that would actually have a major impact on public health (PPPE-I)”.

Some participants believed that with the implementation of the rules, the number of ADRs shall increase to contribute to the global pharmacovigilance system, and a good enforcement mechanism shall be in place. One participant thought that if few regulatory decisions will be taken based on reported ADRs, then definitely public health will be affected by these rules. The public will be gradually aware that their reports have an effect on the regulatory system in Pakistan.

“In Pakistan, many rational formulations were registered in the past which do not exist in the stringent regulatory authorities. But they continue because there is no established ADR reporting system in Pakistan, but after these rules, if the ADRs are reported there might be some deregistration cases (FGS II)”.

A participant from the multinational pharmaceutical industry thought that rules will be just another bureaucratic layer over the system, while another believed that because of their inability to comply with the requirements of the rules, the industry will backlash.

One of the academic pharmacists’ opinions is that it will have a great impact on prescribing, dispensing, and administration of medication, health outcomes of the patient, as well as it will increase patients’ confidence in the healthcare system.

### Theme 5: Coordination, Stakeholder Engagement, and Communication

The majority of the participants thought that the collaboration between DRAP and stakeholders was not at an optimal level. Participants believed that inappropriate selection of stakeholders, lack of coordination between various regional and national pharmacovigilance centers, limited representation of stakeholders from civil society, and lack of understanding and where and how to report ADRs are some of the potential barriers.

“The coordination is far from ideal or the desired level. And the main reason for that I don't blame anyone for that I can see that the DRAP does not have the required manpower and resources where they can outreach and contact the stakeholders and have more frequent interaction with the stakeholders (APhy-I)”.

Recently, DRAP has demonstrated an active role and conducted a series of seminars and training sessions for stakeholders other than healthcare professionals and patients because they are informed through the safety alerts. For some participants, the coordination between DRAP and stakeholders is good. A pharmacist from an international health organization said, in the recent past, DRAP is very active in coordinating with the stakeholders (IHO-I). There is a need for a coordination mechanism within the provinces and hospitals.

For some participants, the coordination between DRAP and stakeholders is not friendly. An academic physician understands that DRAP is facing a shortage of manpower and other resources. A participant from the provincial government service informed that there are two or three drug information centers, and all are in the private sector. In the absence of the DIC, how anyone could contact DRAP for the information remains unknown. The participants other than DRAP were asked about their experience in contacting DRAP for safety information. Most stakeholders were satisfied with their personal experience in contacting DRAP, but they think that it cannot be generalized.

“If you ask my personal experience, it has always been blurred, I could always reach out, but I don’t think that is something that I would say across the board (PI-I)”.

The participants who work with the government were asked to share their experience of contacting the industry for safety information. A federal government official stated that it was a bad experience.

“…There was a manufacturer from which I needed some information on the vaccine safety and I contacted that particular manufacturer, but they were not able to collect the data because they were not collecting that data from the endpoints. So, their vaccine was distributed in the government sector as well as in the public sector. However, they had this whole system on the paper, but it was not implemented. And the reason for being not implemented is that there was no regulatory binding on it. So, this was a bad experience (FGS-I)”.

The participants were asked to identify the key stakeholders to get engaged in the improvement of pharmacovigilance. The majority of the stakeholders proposed to involve multiple stakeholders including DRAP, PHPs, provincial governments, pharmaceutical physicians, district health officers or someone who has control over hospitals, healthcare commission, medical specialized associations, international health partners, journalists, media, and religious leaders. One participant recommended that “we should convince the doctors first. And we should convince the heads of the medical institutions. Either private or public” (AP-II), while another suggested conducting continuous consultative meetings.

“The person who confronts the patients, who is involved with the drugs, the patient? Who really interact with the key stakeholder? who are the key stakeholder the nurse, doctor, and the patients. He should be involved, somebody there who’s a day in and day out dealing with the drugs and the patients and the customers and the consumers (PE-III)”.

Few participants found gaps in the stakeholder’s selection during the development of the pharmacovigilance system. A pharmacist from an international health agency believed that the stakeholder’s selection for the pharmacovigilance system is limited, and the civil society is not involved in the process.

“I believe that the civil society’s role is very important. Fortunately, it’s not, you know, the representation is very limited, although I do agree, we have, you know, representation, but it needs to be expanded (IHO-I)”.

An academic pharmacist argued that media is another stakeholder as far as patients and consumer rights are concerned. The participants from pharmacy academia were of the view that policymakers do not consider them as stakeholders.

“…the only stakeholder they see is the pharmaceutical industry, which is very unfortunate. They need to broaden the understanding of stakeholders and the biggest stakeholder is the consumer, is the patient you need to go back then the nurse then the pharmacist than the doctor(AP I)”.

The key role of regulatory authority in promotion of the public health was described by many participants. An academic pharmacist found DRAP in a dilemma between promoting public health, as well as the goals of the pharmaceutical industry.

One of the inherent issues in our drug regulatory system is that DRAP was really struggling between the two camps. One is public health, and the second is regulating and promoting the pharmaceutical industry. My view is that the DRAP should take the role of somebody who is responsible for public health and not somebody who is promoting the pharmaceutical industry. (AP-I).

The participants who were pharmacists proposed that the pharmacovigilance center should be independent of DRAP and be placed in research or academia settings.

“…It should be independent of DRAP because DRAP is looking at the registration, and licensing of a product. The regulator is doing its own monitoring. My view is that it should be an independent function of from DRAP (AP-I)”.

If I were a doctor, I would not like to talk anything negative about what has happened to my patients. And particularly, they will not like to tell to DRAP. So DRAP is a regulator. That’ is the police. Now, how would a doctor like to talk about an offense to the police? (PPPE-I).

“National and provincial committees on pharmacovigilance can serve as a think-tank on pharmacovigilance and arrange more advocacy sessions. Few participants suggested engaging media and celebrities disseminate the information”.

The academic and practicing pharmacists thought that community pharmacies have a bigger role in educating and also in reporting ADRs. If community pharmacists are offered incentives the number of ADR reports can be increased. Another academic pharmacist and a physician from the pharmaceutical industry recommended incentives and recognition certificates to doctors and heads of hospitals.

“Just a simple sign (Board), If I take this medication, if you take this medication, you experience any good or bad thing, kindly come and talk with your pharmacist or come or talk with this patient (AP-I)”.

### Theme 6: Medicine Safety and Role of Media

There was a unanimous consensus over the role of media in promoting medicine safety. The participants believed that media can be involved through news briefings, writing articles, arranging talk shows, cartoon commercials, social mobilization campaigns, medicine safety campaigns, advertisements, commercials, and dramas to create awareness and dissemination of information. An academic pharmacist emphasized the need to train the media persons with the right knowledge. The role of cartoon journalism and cartoon stories to promote medicine safety was highlighted by both academics from the pharmacy.

“If media can spread the political awareness, why not the medical awareness and why not about the ADRs (APhy-I)”.

Not everyone sees the positive role of media. The participant from the public health program and the pharmaceutical industry highlighted the negative role of the media.

The participants suggested choosing the right media for medicine safety whether it is electronic media, print media, and social media. One of the participants preferred social media over others because it is popular and free. Another participant suggested placing information banners at the different OPDs of the hospitals. One of the participants suggested that the DRAP should have a strong communication team to spread safety information.

## Discussion

The study set out to explore the views of stakeholders on the ADR reporting system of Pakistan, issues with policy, and coordination among stakeholders. The majority of the participants consider the pharmacovigilance system of Pakistan evolving but it is in its infancy. To see if it has an impact, it must get a foothold in the system and start to build roots. Similar findings were observed in the study by Kiguba et al., (2022) on pharmacovigilance in low- and middle-income countries. This is when compared with the high-income countries, the majority of low- and middle-income countries’ regulatory pharmacovigilance systems are nascent or nonexistent.

Often concerns are raised regarding DRAP for poor ADR reporting in the country (Hussain et al., 2018; Atif et al., 2020). Although during 2017–19 the number of ADR reports was not as expected (Khan et al., 2022); however, more than 50,000 adverse events following immunization (AEFIs) reports related to the COVID-19 vaccine have been received by DRAP in the last 2 years. Many participants acknowledged the efforts put in place by the DRAP to improve medicine safety, especially during the COVID-19 pandemic. Due to a shortage of trained staff and the absence of a causality assessment committee, the analysis of received reports is another challenge for the regulatory authority. Pakistan’s pharmacovigilance system is facing the challenges of budgetary constraints, and there is some support from international organizations (Junaidi, 2021). Similar findings were recorded in a study that most LMIC face financial issues, and they rely on the donor’s support (Kiguba et al., 2022).

The participants identified a lack of regulatory framework i.e., pharmacovigilance rules as the major gap in the ADR reporting system which is similar to the findings of a recently conducted quantitative study (Khan et al., 2022). Stakeholders also stated other gaps which include lack of integration among the various components of the health system including hospitals, pharmacies, lack of awareness and knowledge gap, communication gaps between doctors and pharmacists, absence of a causality assessment committee, and the incapacity of the national pharmacovigilance center to evaluate individual case safety reports. Various other studies have mentioned the same gaps in the ADR reporting system of Pakistan (Hussain et al., 2018; Atif et al., 2020; Khan et al., 2022).

Underreporting is generally considered a key limitation to any pharmacovigilance system. Few stakeholders recognized that reporting is discouraged when the reporter does not receive any feedback from the pharmacovigilance center. A randomized study conducted in Sweden explains that feedback from the doctor influences the ADR reporting rate (Wallerstedt et al., 2007). Various other studies also support this notion that the ADR reporting rate is affected by the feedback and some reporters require personal response (Oosterhuis et al., 2011; Rolfes et al., 2015; Al Dweik et al., 2017). One of the challenges discussed by the participants was that there is no active listening going on regarding medicines’ safety. Paying attention to patient voices in vaccine safety has drawn the attention of the researchers. It involves active listening techniques to understand how others assess and perceive risk, and then use this information to empower better decision making (Holt et al., 2016). In response to a question, a participant gave feedback that pharmaceutical companies are reluctant to implement a pharmacovigilance system since this activity does not create profit. A study expressed that in Europe, pharmacovigilance infrastructure is becoming increasingly established, and the high cost of its implementation is being borne by drug manufacturers (Milmo, 2014).

Fear of punitive action among all stakeholders surpasses all barriers to reporting ADRs. Several studies stated the same factors that contribute to hurdles related to ADRs reporting (Al Dweik et al., 2017; Ali et al., 2021; Sharif et al., 2022). The quality of the language and completeness of reports can impede the understanding of the ADR (Hazell and Shakir, 2006). It was also discussed that the English language is one of the barriers among the Pakistani population. The long ADR forms are not in the same language which patients, their attendants, and few healthcare professionals understand these forms. Pakistan may provide ADR reporting forms in regional languages as the Indian pharmacovigilance center has provided consumer reporting forms in 10 languages to tackle the language barrier in ADR reporting (Kalaiselvan et al., 2015).

The pharmaceutical industry is often accused of unethical promotion of medicines. Marketing drugs to physicians including sponsored medical conferences may influence their perception (Kaczmarek, 2022). All stakeholders other than industry representatives also discussed the role of the pharmaceutical industry in profit-making than promoting medicines’ safety. A physician was of the view that the pharmaceutical industry or medical representatives never inform regarding the adverse effects of medicines during medical conferences or personal visits. This has also been observed in the literature (Fickweiler et al., 2017).

To make sure that drug safety monitoring processes are implemented and sustained, the country’s drug regulatory mechanisms should be framed to incorporate pharmacovigilance measures (Alomar et al., 2019). DRAP started consultations on the initial draft of pharmacovigilance rules in 2017. The first draft was prepared in 2018. Since then it has been in the draft format and has not become part of the regulations.

Except for two physicians and one nurse, all participants were aware of the existence of draft pharmacovigilance rules. These findings are similar to what is observed in the literature as studies found that majority of the Pakistani physicians and nurses were not aware of the ADR reporting center and activities in Pakistan (Hussain et al., 2018; 2020a). Some participants have the view that draft rules are as par as international standards. However, some others believed that draft rules are adapted from the international guidelines and not made in the local context and this will not work. Their stance is supported by a study that states that some laws adapted or copied from developed countries are not compatible in the contexts of developing countries (Umeokafor, 2020).

In addition to officers from DRAP, only the representatives of the pharmaceutical industry were aware of the context and contents of the proposed rules. This shows how the pharmaceutical industry in Pakistan watches its interests. There are several studies depicting how the pharmaceutical industry has influenced medicines advertising and promotion in the country (Caudill et al., 1992; Abraham, 2002; Babar et al., 2011; Fugh-Berman and Homedes, 2018; Hailu et al., 2021). One of the pharmacovigilance experts described the policy process as being driven by pharmacists. This is being deduced that the intention of the process is to promote the pharmacists rather than the patient safety.

Generally, politicians initiate policy formulation in areas of major political concern while the permanent bureaucracy has significant power in policy formulation (Buse et al., 2005). Participants believed that bureaucratic red tape and lack of political will are the major reasons for not approving the draft pharmacovigilance rules. According to Bashir’s (2011) analysis, Pakistan’s government sector’s ineffectiveness is mainly due to the high level of red-tapism (Bashir, 2011). Another researcher recommended removing or reducing the red tape from government organizations to improve the efficiency and economy (Rauf, 2020). Various studies support the creation of sustainable budgets for pharmacovigilance staff, routine training, and the development of national pharmacovigilance policies through a political will (Biswas, 2013; Olsson et al., 2015). Political will and sustainability of the pharmacovigilance system are linked in several studies (Abiri and Johnson, 2019). Participants also stated to initiate training and advocacy sessions to convince the political leadership and bureaucracy to bring the pharmacovigilance on agenda and get the rules approved and implemented. A similar framework for communication among doctors, pharmaceutical companies, patients, and DRAP is required. This is similar to what is being developed by the researchers from the Royal College of Physicians of London (RCP) (Allan, 2009).

According to an academic pharmacist, DRAP is attempting to promote public health and the pharmaceutical industry at the same time. A similar observation was shared in a study that states that support from the government for the pharmaceutical industry have not had a positive impact on the quality of medicines. Balance must be established between public health objectives and economic interests. The pharmacy academia suggested placing the national pharmacovigilance center in any academic clinical institution instead of DRAP. They have the view that DRAP is issuing licensing of medicines, hence monitoring of medicines’ side effects would be a conflict; however, this does not hold much substance. Also, the WHO recommends that for a pharmacovigilance center, a government health authority or drugs regulatory agency is the place to govern or establish a pharmacovigilance center.

The COVID vaccine rollout has enhanced the value of global coordination among the stakeholders (Naniche et al., 2021). Although DRAP has shown improvement in coordination with stakeholders during the pandemic, it still lacks harmony and collaboration. Previous studies identified the lack of coordination among the stakeholders (Hussain et al., 2020b; Khan et al., 2022). The public health programs (PHPs) in Pakistan are not integrated with DRAP, except for the expanded program for immunization (EPI). The coordination with EPI is improved because EPI was managed by the COVID vaccine rollout program. As soon as pharmacovigilance rules are approved, all public health programs will need to develop pharmacovigilance systems and integrate them with the DRAP’s national center.

It was also observed that the stakeholders’ selection was not uniform during the development of the pharmacovigilance system and drafting rules with limited or no participation of civil society and academia. Before formulating any policy, it is essential to conduct a stakeholder analysis and engage stakeholders (Adenuga et al., 2020). The role of patient organizations in pharmacovigilance has evolved, with many activities that increase member awareness of and involvement in drug safety, but there are still internal and external barriers to their involvement (Edwards and Graedon, 2010). The representation of the civil society or patient groups in the pharmacovigilance system in Pakistan is none or very limited. This might be due to a lack of awareness and a culture of nonparticipation by the patients and consumers.

Media represents and influences societies in both positive and negative ways. A recent study demonstrated that media coverage may lead to increased adverse event reporting. A balanced approach by the media to cover harm caused by medicines is essential (Edwards and Graedon, 2010). In Pakistan, pharmaceutical companies alleged that they are sometimes blackmailed by the media if any incident occurred due to their medicine. This shows the dark side of yellow journalism (Ricchiardi, 2012; Kurambayev, 2017). A similar study showed how media creates hypes in case of mass casualty incidences (Musharraf et al., 2022). Due to the growing popularity of the use of social media the participants also suggested the promotion of medicine safety. The same was suggested by Yasir Al-Worafi (2020) that social media could be used to strengthen the pharmacovigilance systems.

The participants presented a number of strategies to improve the pharmacovigilance system of Pakistan, as presented in recent studies (Hussain et al., 2020a; Atif et al., 2020; Shchory et al., 2020; Bahri and Pariente, 2021; Khan et al., 2022). The participants also proposed that the healthcare practitioners who interact with patients should be involved to improve the pharmacovigilance system in the country. The role of nonpharmacists in community pharmacies is also neglected and they also needed to be brought into the discussion. Community pharmacists can also play a pivotal role in increasing the number of ADR reports. Linking institutional purchases with the availability of pharmacovigilance systems can also improve the culture of pharmacovigilance.

For a robust and functional pharmacovigilance system in Pakistan, the study participants proposed 1) immediate approval of pharmacovigilance rules, 2) training and advocacy sessions to pursue the political leadership and bureaucracy, 3) establishment of a Pharmacovigilance Risk Assessment Expert Committee, 4) recruitment of trained staff, 5) allocation of a separate budget for pharmacovigilance activities, 6) capacity building and integration among the various components of the health system including hospitals, pharmacies, public health programs with provincial or central pharmacovigilance centers, 7) to update medical, pharmacy, and nursing curriculum with the inclusion of pharmacovigilance, 8) involving media to promote medicine safety, 9) involving nonpharmacists at community pharmacies, and 10) conducting local clinical trials to generate local safety data. This is the first ever inductive qualitative study conducted in Pakistan on the ADR reporting system, policy, and coordination involving a broad range of stakeholders. Any review of the pharmacovigilance policy of Pakistan by policymakers can get help from findings from the current study as a crucial component.

### Limitations to the Study

The study sample did not include key informants from individual Pakistani provinces where there is no ADR reporting system in place. These provinces are Sind, Khyber Pakhtunkhwa, Baluchistan, and federally administered areas Gilgit Baltistan and Azad Jammu and Kashmir. Participants’ selection was purposive, and we do not know if the views and experiences of participants who have withdrawn from the study differ from those of their colleagues. Not including patient support groups and media may also have restricted the range of stakeholders.

## Conclusion

The study concluded that the stakeholders were partially satisfied with the progress made with the current pharmacovigilance system. Although the pharmacovigilance rules are available in the draft format, there is a need for the approval of the legal framework. However, before approval and implementation, a wider consultation of multi-stakeholders including the patient groups and journalists will help address the policy issues. Through advocacy and training of stakeholders, removing barriers of red-tape, having a political will, and motivating the willingness of HCPs are the major objectives to be achieved. By engaging stakeholders, technology, and media, the medicines’ safety information can be disseminated to the masses to improve the safety of medicines.

## Data Availability

The raw data supporting the conclusion of this article will be made available by the authors, without undue reservation.
